# Diagnostic Codes in AI Prediction Models and Label Leakage of Same-Admission Clinical Outcomes

**DOI:** 10.1001/jamanetworkopen.2025.50454

**Published:** 2025-12-26

**Authors:** Bashar Ramadan, Ming-Chieh Liu, Michael C. Burkhart, William F. Parker, Brett K. Beaulieu-Jones

**Affiliations:** 1 Center for Computational Medicine and Clinical Artificial Intelligence, Department of Medicine, University of Chicago, Chicago, Illinois; 2 MacLean Center for Clinical Medical Ethics, University of Chicago, Chicago, Illinois

## Abstract

**Importance:**

Artificial intelligence models that predict same-admission outcomes for hospitalized patients, such as inpatient mortality, often rely on *International Classification of Diseases* (*ICD*) diagnostic codes, even when these codes are not finalized until after discharge.

**Objective:**

To investigate the extent to which the inclusion of *ICD* codes as features in predictive models are associated with inflated performance metrics via label leakage (eg, including the code for cardiac arrest into an inpatient mortality prediction model) and assess the prevalence and implications of this practice in existing literature.

**Design, Setting, and Participants:**

This prognostic study examined publicly available, deidentified inpatient electronic health record data from the Medical Information Mart for Intensive Care IV (MIMIC-IV) database. Patients admitted to an intensive care unit or emergency department at Beth Israel Deaconess Medical Center between January 1, 2008, and December 31, 2019, were included. These data were analyzed between December 18, 2024, and January 14, 2025. A targeted literature review of same-admission prediction models using MIMIC with *ICD* codes as features was performed between November 20 and 27, 2024.

**Main Outcome and Measures:**

Using a standard training-validation-test split procedure, prediction models were developed for inpatient mortality (logistic regression, random forest, and XGBoost) using only *ICD* codes as features. Performance in the test set was analyzed using areas under the receiver operating curve and variable importance. Frequencies of studies using same-admission prediction models using MIMIC with *ICD* codes were calculated from the targeted literature review.

**Results:**

The study cohort consisted of 180 640 patients (mean [SD] age at admission, 58.7 [19.2] years; 53.0% female), of whom 8573 (4.7%) died during the admission. The models using *ICD* codes predicted in-hospital mortality with high performance in the test dataset, with areas under the receiver operating curve of 0.976 (95% CI, 0.973-0.980) (logistic regression), 0.971 (95% CI, 0.967-0.974) (random forest), and 0.973 (95% CI, 0.968-0.977) (XGBoost). The most important *ICD* codes were subdural hemorrhage (OR, 389.99; 95% CI, 28.79-5283.59), cardiac arrest (OR, 219.58; 95% CI, 159.61-302.08), brain death (OR, 112.78; 95% CI, 13.42-947.70), and encounter for palliative care (OR, 98.04; 95% CI, 83.16-115.58). The literature review found that 37 of 92 studies (40.2%) using MIMIC to predict same-admission outcomes included *ICD* codes as features, even though both MIMIC publications and documentation clearly state that *ICD* codes are derived after discharge.

**Conclusions and Relevance:**

This prognostic study of the MIMIC-IV database suggests that using *ICD* codes as features in same-admission prediction models may be a severe methodological flaw associated with inflated performance metrics, rendering models incapable of clinically useful predictions. The literature review found that the practice is common. Addressing this challenge is essential for advancing trustworthy artificial intelligence in health care.

## Key Points

**Question:**

Are *International Classification of Diseases* (*ICD*) diagnostic codes, which are only finalized after hospital discharge, associated with inflated performance of artificial intelligence (AI) health care prediction models?

**Findings:**

In this prognostic study of 180 640 patients, 40.2% of published AI models trained to predict same-admission outcomes used *ICD* codes as features. Prediction models for inpatient mortality trained on *ICD* codes predicted in-hospital mortality with high accuracy, with the most important codes (eg, brain death, encounter for palliative care) not available in time for clinically useful mortality prediction.

**Meaning:**

These findings suggest that to ensure that AI prediction models are both reliable and clinically deployable, greater diligence is needed in identifying and preventing label leakage.

## Teaser

This prognostic study investigates whether artificial intelligence (AI) prediction models for in-hospital mortality using *International Classification of Diseases* diagnostic codes are associated with inflated performance metrics via label leakage.

## Introduction

Artificial intelligence (AI) and machine learning models have shown impressive performance in predicting critical same-admission outcomes, such as in-hospital mortality.^1,2,3^ Some models use *International Classification of Diseases* (*ICD*) diagnostic billing codes as input features. Since *ICD* codes are entered in the electronic health record (EHR) after a clinical event, can be revised over the course of an admission, and are finalized only after discharge, their inclusion introduces data leakage, in which information unavailable in deployed clinical settings is improperly used during model training and evaluation.

There are many published examples of machine learning models in health care achieving unrealistic performance by relying on unintended features, a phenomenon termed shortcut learning.^4,5,6,7,8^ In this work, we specifically examined the issue of temporal label leakage, as described by Davis et al,^9^ in which model inputs are used before they are actually available. For example, imagine a patient admitted with unspecified abdominal pain. After further evaluation, the patient is diagnosed with appendicitis, develops septic shock, and experiences cardiac arrest several days later before dying. Early in the patient’s admission, only unspecified abdominal pain would be available. However, if a model incorporates all *ICD* codes subsequently assigned after the end of a hospital stay, it unfairly leverages hindsight information to predict mortality, achieving deceptively high accuracy.

This work aimed to illustrate how seemingly accurate same-admission prediction models may be driven by leakage and to quantify how frequently such leakage appears in the literature on machine learning for health care. To examine outcomes associated with this problem, we performed 2 analyses. First, we use *ICD* codes in models predicting inpatient mortality, one of the most common same-admission prediction tasks. Second, we performed a targeted literature review of studies that have built AI models to predict inpatient outcomes and identified the percentage of those that included *ICD* codes from the same admission as input features.

## Methods

### Data Source and Study Population

This prognostic study used the Medical Information Mart for Intensive Care IV database (MIMIC-IV), version 2.2,^10^ a publicly available, deidentified, EHR database of patients admitted to an intensive care unit (ICU) or emergency department at Beth Israel Deaconess Medical Center between January 1, 2008, and December 31, 2019. The MIMIC-IV database is a large, freely accessible EHR resource released in deidentified form, with dates shifted and other deidentification safeguards applied per Health Insurance Portability and Accountability Act deidentification standards. Because the research used only deidentified data and involved no interaction with individuals, no access to identifiable private information, and no intervention, it did not constitute human participants research under the Common Rule and, therefore, did not require institutional review board review or informed consent. Access to the MIMIC data followed standard credentialing requirements and data use agreement. This study followed the Transparent Reporting of a Multivariable Prediction Model for Individual Prognosis or Diagnosis and AI (TRIPOD+AI) and Strengthening the Reporting of Observational Studies in Epidemiology (STROBE) reporting guidelines.

All admissions with *ICD* codes were included in our study, with less than 1% excluded. The MIMIC-IV dataset categorizes race and ethnicity data of admitted patients as Asian, Black, Hispanic, White, other, or unknown, which are reported herein for descriptive purposes.^10^ We partitioned the dataset by the date of admission into train (70%), validation (10%), and test (20%) sets per TRIPOD+AI guidelines,^11^ excluding patients from the validation and test sets who also had admissions in the training set. Because our cohort spans the US transition from The *International Classification of Diseases, Ninth Revision, Clinical Modification* (*ICD-9-CM*) to the *International Statistical Classification of Diseases, Tenth Revision, Clinical Modification* (*ICD-10-CM*) (October 1, 2015), we mapped all *ICD-10-CM* diagnoses to *ICD-9-CM* using the Centers for Medicare & Medicaid Services General Equivalence Mappings to harmonize the code space across years; the additional granularity of *ICD-10-CM* was not required for our aims focused on leakage. We also removed *ICD* codes that had low variance (<0.0001) or high covariance (>0.8) with other *ICD* codes.

### *ICD* Code Prediction Model Development and Evaluation

We trained classification models (logistic regression,^12^ random forest,^12^ and XGBoost^13^) using only *ICD-9* codes as features, tuning hyperparameters in the validation set. We chose these models because they are some of the commonly used classifiers, achieve strong performance with tabular data, and offer approaches to interpret models. Other predictive features, such as vital signs, laboratory values, and medications, were intentionally excluded to examine only the potential for *ICD* code–driven label leakage. The trained models were then evaluated on the held-out test set, with performance assessed using the area under the receiver operating characteristic curve (AUROC) and balanced accuracy.

### Targeted Literature Review

To assess the pervasiveness of this issue, we performed a targeted literature review of studies that used either MIMIC-III or MIMIC-IV. To do so, we used Google Scholar between November 20 and 27, 2024, with 2 search queries (case insensitive): (1) *prediction model machine learning mimic-IV* OR *mimic IV* OR *mimic 4* OR *mimic-4* and (2) *prediction model machine learning mimic-III* OR *mimic III* OR *mimic 3* OR *mimic-3*. We sorted results by citations per year to avoid bias against recently published studies and screened them sequentially until we identified 100 prediction modeling studies (50 each for MIMIC-III^14^ and MIMIC-IV^10^). We then performed a manual review of the articles to (1) categorize whether the studies predicted clinical events during the same admission and (2) investigate whether *ICD* codes were used as input features to predict an outcome during that same admission.

### Statistical Analysis

We calculated odds ratios (ORs) and *P* values for *ICD* codes in the logistic regression model and applied the Benjamini-Hochberg procedure to control for false discovery rate, with a threshold of *P* < .05. For the random forest and XGBoost models, we assessed feature importance with each library’s respective default criterion, namely Gini importance and gain, to identify which *ICD* codes were considered important for the prediction task. The analysis was performed between December 18, 2024, and January 14, 2025, using Python, version 3.10 (Python Software Foundation) with the packages numpy, version 2.0.2; pandas, version 2.2.2; scikit-learn, version 1.4.2; scipy, version 1.13.0; shap, version 0.46.0; statsmodels, version 0.14.2, and xgboost, version 2.0.3. The full source code is available on Github.^15^

## Results

### *ICD* Code–Based Prediction Models

**Figure 1.**
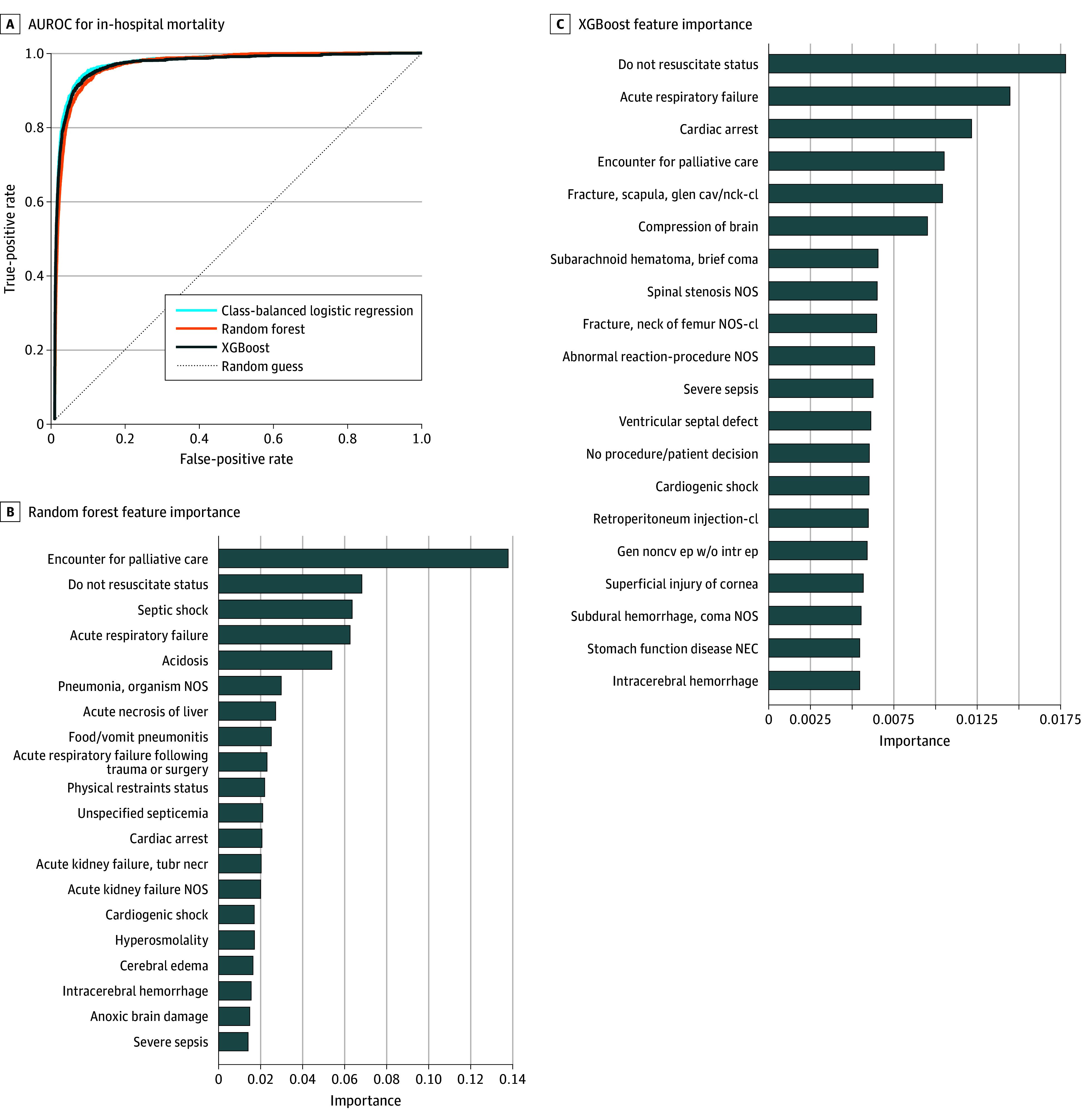
Model Predictive Performance and Feature Importance for Predicting In-Hospital Mortality A, Shading indicates the 95% CI. AUROC indicates area under the receiver operating characteristic curve; cl, closed; gen noncv ep w/o intr ep, generalized nonconvulsive epilepsy without mention of intractable epilepsy; glen cav/nck, glenoid cavity and the scapular neck; NEC, necrotizing enterocolitis; NOS, not otherwise specified; tubr necr, tubular necrosis.

The study cohort included 422 534 hospital admissions from 180 640 unique patients (mean [SD] age at admission, 58.7 [19.2] years; 53.0% female and 47.0% male; 3.5% categorized in MIMIC-IV as Asian, 16.2% as Black, 5.9% as Hispanic, 67.2% as White, 4.1% as other, and 3.3% as unknown race and ethnicity). In-hospital mortality occurred in 8417 admissions (2.0%). In the held-out test set, all 3 models achieved high predictive performance, with AUROCs of 0.976 (95% CI, 0.973-0.980) (logistic regression), 0.971 (95% CI, 0.967-0.974) (random forest), and 0.973 (95% CI, 0.968-0.977) (XGBoost) (Figure 1A; eFigure in Supplement 1). These results are even better than published models trained on the same data that also included many additional predictive features from the rest of the EHR.^1,2^

The Table highlights the 20 diagnostic codes with the highest ORs used by the logistic regression model. Complete logistic regression feature results are available in eTable 2 in Supplement 2. All codes were statistically significant after the Benjamini-Hochberg procedure (*P* < .05). Acute diagnoses typically arose during hospitalization and dominated the list, such as subdural hematoma, deep coma (OR, 389.99; 95% CI, 28.79-5283.59); cardiac arrest (OR, 219.58; 95% CI, 159.61-302.08); brain death (OR, 112.78; 95% CI, 13.42-947.70); and encounter for palliative care (OR, 98.04; 95% CI, 83.16-115.58), all of which carry an obvious high risk of mortality. The additional features included rare diagnoses and symptoms that occurred in cases more often than controls within this dataset.

**Table.** Top 20 Features in the Logistic Regression Model

Feature	OR (95% CI)	Adjusted *P* value^a^
Subdural hemorrhage, deep coma	389.99 (28.79-5283.59)	<.001
Cardiac arrest	219.58 (159.61-302.08)	<.001
Brain death	112.78 (13.42-947.70)	<.001
Encounter for palliative care	98.04 (83.16-115.58)	<.001
Transient visual loss	96.12 (45.58-202.69)	<.001
Kidney sclerosis, unspecified	69.83 (43.92-111.03)	<.001
Unspecified intracranial hemorrhage	59.52 (32.96-107.48)	<.001
Acute maxillary sinusitis	37.24 (12.36-112.17)	<.001
Chronic glomerulonephritis with unspecified pathologic lesion in kidney	36.30 (15.77-83.56)	<.001
Subarachnoid hemorrhage following injury without mention of open intracranial wound, with prolonged (>24 h) loss of consciousness without return to preexisting conscious level	32.08 (2.23-461.70)	.04
Abdominal aneurysm, ruptured	30.90 (14.75-64.72)	<.001
Postoperative shock, cardiogenic	28.64 (15.67-52.37)	<.001
Influenza due to identified avian influenza virus with other respiratory manifestations	26.00 (11.72-57.69)	<.001
Other abnormality of urination	25.95 (13.06-51.54)	<.001
Intracerebral hemorrhage	25.85 (21.83-30.60)	<.001
Nonpressure chronic ulcer of other part of right foot with other specified severity	25.70 (6.97-94.78)	<.001
Ulcer of thigh	22.75 (9.76-53.02)	<.001
Acute myeloid leukemia, in relapse	21.79 (13.21-35.94)	<.001
Viral hepatitis B with hepatic coma, acute or unspecified, without mention of hepatitis delta	21.68 (7.21-65.18)	<.001
Unspecified drug dependence, unspecified	20.96 (7.81-56.27)	<.001

Abbreviation: OR, odds ratio.

^a^ Benjamini-Hochberg correction.

Feature importance analyses from the random forest and XGBoost models (Figure 1B) found *ICD* codes for do not resuscitate status (random forest rank, 2nd; XGBoost rank, 1st), acute respiratory failure (random forest rank, 4th; XGBoost rank, 2nd), and encounter for palliative care (random forest rank, 1st; XGBoost rank, 4th) to be powerful predictors of mortality. In addition to *ICD* codes that obviously represent label leakage (eg, brain death), the diagnosis superficial injury to the cornea was the 17th most important feature to the XGBoost model, which stood out as it is not an acute diagnosis. This anomaly may be associated with the model’s ability to detect a clinician’s focus on documenting less severe conditions, signaling relative patient stability and, therefore, low mortality risk.

### Literature Review

**Figure 2.**
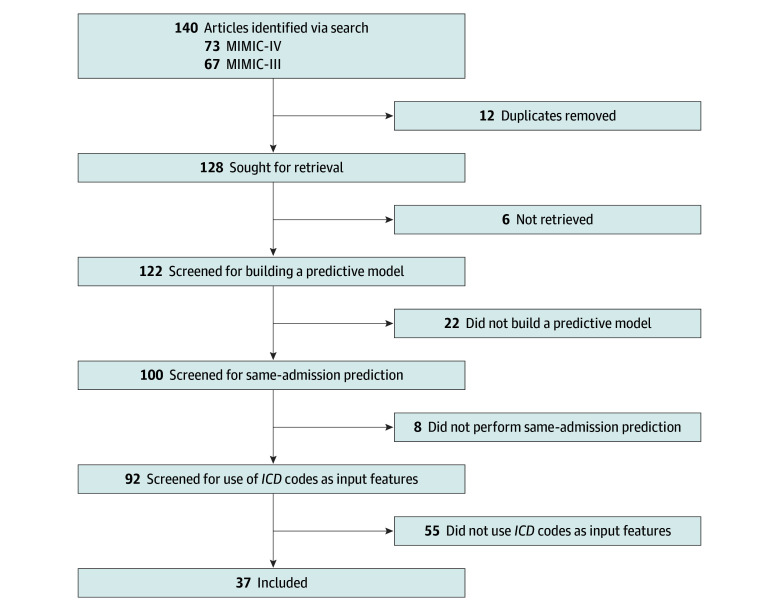
Overview of Targeted Literature Screening and Review The screening involved searching and filtering studies citing Medical Information Mart for Intensive Care III (MIMIC-III) or MIMIC-IV, developing a prediction model, performing same-admission predictions, and using *International Classification of Diseases* (*ICD*) codes as input features.

Figure 2 outlines our study-screening process. We reviewed 100 studies that built a prediction model from an initial set of the 140 citing MIMIC and sorted them in descending order by the mean number of citations per year (the full list is provided in eTable 1 in Supplement 1).^16,17,18,19,20,21,22,23,24,25,26,27,28,29,30,31,32,33,34,35,36,37,38,39,40,41,42,43,44,45,46,47,48,49,50,51,52,53,54,55,56,57,58,59,60,61,62,63,64,65,66,67,68,69,70,71,72,73,74,75,76,77,78,79,80,81,82,83,84,85,86,87,88,89,90,91,92,93,94,95,96,97,98,99,100,101,102,103,104,105,106,107,108,109,110,111,112,113,114,115^ Of these articles, 92 (92.0%) reported building predictive models that targeted outcomes within the same admission,^17,18,19,21,22,23,24,25,26,27,28,29,30,31,32,33,34,35,36,37,38,39,40,41,42,43,44,45,46,47,48,49,50,51,52,53,54,55,56,57,58,59,60,62,63,64,65,67,68,69,70,71,72,73,74,75,76,77,78,79,82,83,84,85,86,87,88,89,90,91,92,94,95,96,97,98,99,100,101,102,103,104,105,106,107,109,110,111,112^ and among those, 37 (40.2%) used *ICD* diagnostic codes as input features.^17,21,22,26,30,34,35,38,40,41,42,43,47,49,50,58,59,60,68,70,71,75,78,83,84,87,90,92,97,98,99,102,103,107,109,111,115^

## Discussion

This prognostic study found that a specific problem within the machine learning health care literature may be the presence of data leakage in same-admission prediction models associated with the inclusion of diagnostic codes as input features. These codes, finalized only after discharge, provide models with hindsight information that would not be available at the time of prediction. This practice causes 2 distinct and serious problems. First, codes that clinicians document in the EHR after a clinical encounter cannot be used to guide real-time clinical decision making during that encounter. Second, a subset of these codes (eg, brain death for inpatient mortality) document highly correlated events with the outcome being predicted. This issue underscores a broader concern that machine learning models trained with retrospective data risk misrepresenting their value in actual clinical care. If these models do not account for the realities of real-time clinical workflows, their success in research will not translate into meaningful improvements in patient outcomes.

Both MIMIC-III and MIMIC-IV carry explicit warnings against using an admission’s *ICD* codes to predict outcomes from that same admission. In MIMIC-III, *ICD-9* codes arise from patient discharges,^14^ while MIMIC-IV clarifies that diagnoses are determined by trained professionals after reviewing signed patient notes.^10^ These datasets do not provide an audit log of changes or updates to *ICD* codes but, instead, provide only the final set of *ICD* diagnoses. Given the prevalence of *ICD* code use in MIMIC-based studies despite this direct guidance, we suspect that publications on private institutional data, especially those that do not share source code, could potentially be even more likely to be compromised by label leakage.

Researchers aim to harness available knowledge to the greatest extent possible when training models, and there is a reasonable expectation that some diagnoses are known to clinicians shortly after admission (eg, broken limbs, burns). Some information could potentially be gleaned from patient notes or physician problem lists that may be available during a patient’s stay. Often, codes are carried over from previous visits, eg, chronic conditions or comorbidities such as diabetes and hypertension, and these can safely be assumed as known. However, diagnoses in the form of *ICD* codes for a given admission in MIMIC are explicitly derived after discharge. In other datasets, it may be possible to use *ICD* codes without label leakage if these codes are time-stamped and derived from problem lists. However, there are still substantial limitations given that these codes are used for billing purposes and represent clinical thinking as opposed to patient state.^3^

Both analyses in this study have a scope limited to the MIMIC dataset. However, thousands of studies have used data from the MIMIC database^10,14^ for a wide variety of tasks, including the portion incorporating *ICD* codes for same-admission prediction tasks identified in this study. While it is not possible to quantify this issue for private or institutional datasets, we suspect that similar issues may be at least as prevalent in analyses on less transparent and thoroughly documented datasets. The MIMIC database is well described, with detailed publications, well-developed documentation, and example code for analyses. Institutional and private datasets generally have less transparency and do not allow for reproducibility, reflecting a broader challenge in health care machine learning research.^9^ That label leakage occurs this often in a well-defined dataset that explicitly describes the nature of *ICD* codes should raise questions when evaluating research using less transparent datasets and methods.

A solution to the problem of temporal label leakage is to diligently examine the input features to ensure that these features are truly available at the time of prediction, which could be a challenging problem in health care due to the complicated nature of data generation. For example, present-on-admission flags seem like an easy way to decide whether an *ICD* code could be used in same-admission prediction. In reality, the Centers for Medicare & Medicaid Services states that “subsequent to the assignment of the *ICD-10-CM* codes, the [present-on-admission] indicator should then be assigned to those conditions that have been coded.”^116^ There are many examples of apparent timestamps, which are actually imperfect proxies for when information is known because of the way documentation lags clinical reality. Accordingly, our recommendation is to ensure that model developers are only using data based on the EHR storage time as opposed to either making assumptions about availability or using other timing information. Model developers could visualize the passage of time with patient timelines based on the EHR storage time to emulate the clinical deployment of prediction models. It is critical for research teams to work with clinical domain experts, as well as information technologists and informaticians, to understand the meaning of different timestamps in clinical data. We advise defining the prediction time point a priori and, for any candidate variable, establishing whether it is truly known by that moment through provenance review and clinician or domain-expert input. We also recommend that articles include a brief variable availability statement that names the source and timing assumptions for each variable class and explains how those assumptions align with the intended clinical use.

The utility of *ICD* codes geared at billing for deployable prediction models is debatable, but at a minimum, researchers need to be careful to ensure that the codes are available prior to the time a prediction needs to be made. Ensuring codes would be available may require only using codes from prior admissions, which still requires ensuring that they are not edited during any adjudication processes with payers or deriving these diagnoses from a time-stamped problem list. The MIMIC database, however, does not include either timestamps or codes from the problem list. The frequency of this error suggests a need for researchers to more closely read the documentation of third-party datasets. While it is not possible to estimate how frequently this issue occurs in private, institutional datasets, we believe that the frequency also suggests a need for greater engagement of prediction model developers with experts covering the full data generation (clinicians) and preparation (eg, informaticians and data warehousing teams) process.

### Limitations

This study had some limitations. The scope was limited to studies that used the MIMIC-III and MIMIC-IV datasets. Our findings suggest a clear problem within this subset of the literature but did not provide direct evidence of whether or how frequently this issue occurs in studies that used private or other institutional datasets. Furthermore, this analysis did not account for potential differences between MIMIC and private institutional data, which may have different coding practices, data structures, or documentation. While we suspect that similar or greater challenges may exist in less transparent datasets because of their less transparent nature, it is not possible to empirically test this. Thus, this study includes no findings to support that assertion. Any generalization of our findings beyond the MIMIC-based literature would require further investigation.

## Conclusions

This prognostic study of patient data in the MIMIC-IV database found that using *ICD* codes as features in same-admission prediction models may be a severe methodological flaw that inflates performance metrics and renders models incapable of making clinically useful predictions in real time. Our literature review found that the practice is common. Addressing this challenge is essential for advancing trustworthy AI in health care.

## Notes

Supplement 1. **eTable 1.** Studies Citing MIMIC That Built a Predictive Model **eFigure.** Calibration Curves and Predicted Distributions for Trained Classifiers
eReferences.

Supplement 2. **eTable 2.** Full List of Features’ Odds Ratios for the Logistic Regression Model

Supplement 3.
Data Sharing Statement
