# Supplementary material for: Diagnostic Codes in AI Prediction Models and Label Leakage of Same-Admission Clinical Outcomes
Source: JAMA Netw Open. 2025 Dec 26;8(12):e2550454. doi: 10.1001/jamanetworkopen.2025.50454 (PMC12743287; doi:10.1001/jamanetworkopen.2025.50454)
Supplement: Supplement 1. — eTable 1. Studies Citing MIMIC That Built a Predictive Model eFigure. Calibration Curves and Predicted Distributions for Trained Classifiers eReferences. [file jamanetwopen-e2550454-s001.pdf]

Supplemental Online Content 1

Ramadan B, Liu MC, Burkhart MC, Parker WF, Beaulieu-Jones BK. Diagnostic codes in AI prediction models and label leakage of same-admission clinical outcomes. *JAMA Netw Open*. 2025;8(12):e2550454. doi:10.1001/jamanetworkopen.2025.50454

eTable 1. Studies Citing MIMIC That Built a Predictive Model
eFigure. Calibration Curves and Predicted Distributions for Trained Classifiers
eReferences

This supplemental material has been provided by the authors to give readers additional information about their work.

© 2025 Ramadan B et al. *JAMA Network Open*.

eTable 1. Studies Citing MIMIC That Built a Prediction Model

Title	Pub status	Mimic 3 v 4	Same admission prediction?	ICD codes used as features?	Year
Predicting 30-days mortality for MIMIC-III patients with sepsis-3: a machine learning approach using XGboost[1]	Journal (J Transl Med)	III	FALSE	FALSE	2020
Prediction model of in-hospital mortality in intensive care unit patients with heart failure: machine learning-based, retrospective analysis of the MIMIC-III database[2]	Journal (BMJ Open)	III	TRUE	TRUE	2021
Predicting sepsis with a recurrent neural network using the MIMIC III database[3]	Journal (Comput Biol Med)	III	TRUE	FALSE	2019
Machine-learning models for prediction of sepsis patients mortality[4]	Journal (Med Intensiva)	IV	TRUE	FALSE	2023
Machine learning prediction models for prognosis of critically ill patients after open-heart surgery[5]	Journal (Sci Rep)	III	FALSE	TRUE	2021
A machine learning-based prediction model for in-hospital mortality among critically ill patients with hip fracture: An internal and external validated study[6]	Journal (Injury)	III	TRUE	TRUE	2023
Machine learning prediction models for mechanically ventilated patients: analyses of the MIMIC-III database[7]	Journal (Front Med)	III	TRUE	TRUE	2021
Towards a decision support tool for intensive care discharge: machine learning algorithm development using electronic healthcare data from MIMIC-III and Bristol, UK[8]	Journal (BMJ Open)	III	TRUE	FALSE	2019
Development and validation of a machine-learning model for prediction of extubation failure in intensive care units[9]	Journal (Front Med)	IV	TRUE	FALSE	2021
Benchmarking emergency department prediction models with machine learning and public electronic health records[10]	Journal (Sci Data)	IV	TRUE	FALSE	2021
Application of interpretable machine learning for early prediction of prognosis in acute kidney injury[11]	Journal (Comput Struct Biotechnol J)	IV	TRUE	TRUE	2022
Developing an explainable machine learning model to predict the mechanical ventilation duration of patients with ARDS in intensive care units[12]	Journal (Heart Lung)	IV	TRUE	FALSE	2023
Developing machine learning models for prediction of mortality in the medical intensive care unit[13]	Journal (Comput Methods Programs Biomed)	III	TRUE	FALSE	2022
A novel machine learning model to predict respiratory failure and invasive mechanical ventilation in critically ill patients suffering from COVID-19[14]	Journal (Sci Rep)	III	TRUE	FALSE	2022
Predicting hospital length of stay using neural networks on mimic iii data[15]	Conference (IEEE)	III	TRUE	TRUE	2017
A machine learning-based prediction model for acute kidney injury in patients with congestive heart failure[16]	Journal (Front Cardiovasc Med)	III	TRUE	FALSE	2022
Explainable machine-learning model for prediction of in-hospital mortality in septic patients requiring intensive care unit readmission[17]	Journal (Infect Dis Ther)	IV	TRUE	FALSE	2022
Atrial fibrillation detection during sepsis: study on MIMIC III ICU data[18]	Journal (IEEE J Biomed Health Inform)	III	TRUE	FALSE	2020

© 2025 Ramadan B et al. *JAMA Network Open*.

A machine-learning approach for dynamic prediction of sepsis-induced coagulopathy in critically ill patients with sepsis[19]	Journal (Front Med)	IV	TRUE	TRUE	2020
Prediction of intensive care unit length of stay in the MIMIC-IV dataset[20]	Journal (Appl Sci)	IV	TRUE	TRUE	2023
Early prediction of ventilator-associated pneumonia in critical care patients: a machine learning model[21]	Journal (BMC Pulm Med)	III	TRUE	FALSE	2022
Predicting duration of mechanical ventilation in acute respiratory distress syndrome using supervised machine learning[22]	Journal (J Clin Med)	III	TRUE	FALSE	2021
Prediction of length-of-stay at intensive care unit (ICU) using machine learning based on MIMIC-III database[23]	Conference (IEEE)	III	TRUE	TRUE	2023
Mapping patient trajectories using longitudinal extraction and deep learning in the MIMIC-III critical care database[24]	Journal (Pac Symp Biocomput)	III	TRUE	FALSE	2017
Machine learning prediction models and nomogram to predict the risk of in-hospital death for severe DKA: A clinical study based on MIMIC-IV, eICU[25]	Journal (Int J Med Inform)	IV	TRUE	TRUE	2023
Development of a machine learning-based prediction model for sepsis-associated delirium in the intensive care unit[26]	Journal (Sci Rep)	IV	TRUE	TRUE	2023
Novel pneumonia score based on a machine learning model for predicting mortality in pneumonia patients on admission to the intensive care unit[27]	Journal (Respir Med)	IV	TRUE	TRUE	2023
Predictive modeling in urgent care: a comparative study of machine learning approaches[28]	Journal (JAMIA Open)	III	TRUE	TRUE	2018
Development of a nomogram to predict 28-day mortality of patients with sepsis-induced coagulopathy: an analysis of the MIMIC-III database[29]	Journal (Front Med)	III	TRUE	FALSE	2021
Mortality prediction for patients with acute respiratory distress syndrome based on machine learning: a population-based study[30]	Journal (Ann Transl Med)	III	TRUE	FALSE	2021
Machine learning models for early prediction of sepsis on large healthcare datasets[31]	Journal (Electronics)	III	TRUE	FALSE	2022
Outcome Prediction in Critically-Ill Patients with Venous Thromboembolism and/or Cancer Using Machine Learning Algorithms: External Validation and Comparison with Scoring Systems[32]	Journal (Int J Mol Sci)	III	TRUE	TRUE	2022
Statistical analysis and machine learning prediction of disease outcomes for COVID-19 and pneumonia patients[33]	Journal (Front Cell Infect Microbiol)	III	TRUE	FALSE	2022
Prediction model of in-hospital mortality in intensive care unit patients with cardiac arrest: a retrospective analysis of MIMIC-IV database based on machine[34]	Journal (BMC Anesthesiol)	IV	TRUE	TRUE	2023
Development and assessment of scoring model for ICU stay and mortality prediction after emergency admissions in ischemic heart disease: a retrospective study of MIMIC-IV databases[35]	Journal (Intern Emerg Med)	IV	TRUE	TRUE	2023
Tendency of dynamic vasoactive and inotropic medications data as a robust predictor of mortality in patients with	Journal (Front Cardiovasc Med)	IV	TRUE	FALSE	2023

© 2025 Ramadan B et al. *JAMA Network Open*.

septic shock: An analysis of the MIMIC-IV database[36]					
Benchmarking PySyft federated learning framework on MIMIC-III dataset[37]	Journal (IEEE Access)	III	TRUE	FALSE	2021
Development and validation of a deep learning model to predict the survival of patients in ICU[38]	Journal (J Am Med Inform Assoc)	III	TRUE	FALSE	2022
Establishment of ICU mortality risk prediction models with machine learning algorithm using MIMIC-IV database[39]	Journal (Diagnostics)	IV	TRUE	FALSE	2022
Development and validation of a novel blending machine learning model for hospital mortality prediction in ICU patients with Sepsis[40]	Journal (BioData Min)	III	TRUE	FALSE	2021
Machine learning algorithms for prediction of ventilator associated pneumonia in traumatic brain injury patients from the MIMIC-III database[41]	Journal (Heart Lung)	III	TRUE	FALSE	2023
Construction and validation of machine learning models for sepsis prediction in patients with acute pancreatitis[42]	Journal (BMC Surg)	III & IV	TRUE	FALSE	2023
A machine learning–based algorithm for the prediction of intensive care unit delirium (PRIDE): retrospective study[43]	Journal (JMIR Med Inform)	III	TRUE	TRUE	2021
Dendrogram of transparent feature importance machine learning statistics to classify associations for heart failure: A reanalysis of a retrospective cohort study of the Medical Information Mart for Intensive Care III (MIMIC-III) database[44]	Journal (PLoS One)	III	TRUE	TRUE	2023
Mortality prediction among ICU inpatients based on MIMIC-III database results from the conditional medical generative adversarial network[45]	Journal (Heliyon)	III	TRUE	TRUE	2023
Machine learning prediction models for postoperative stroke in elderly patients: analyses of the MIMIC database[46]	Journal (Front Aging Neurosci)	III & IV	FALSE	TRUE	2022
Early predicting 30-day mortality in sepsis in MIMIC-III by an artificial neural networks model[47]	Journal (Eur J Med Res)	III	TRUE	FALSE	2022
Predicting mortality using machine learning algorithms in patients who require renal replacement therapy in the critical care unit[48]	Journal (J Clin Med)	III	TRUE	FALSE	2022
A simple weaning model based on interpretable machine learning algorithm for patients with sepsis: a research of MIMIC-IV and eICU databases[49]	Journal (Front Med)	IV	TRUE	FALSE	2021
Real-time mortality prediction using MIMIC-IV ICU data via boosted nonparametric hazards[50]	Conference (IEEE)	IV	TRUE	FALSE	2021
Critical correlation of predictors for an efficient risk prediction framework of ICU patient using correlation and transformation of MIMIC-III dataset[51]	Journal (Data Sci Eng)	III	FALSE	TRUE	2022
Machine Learning Approach to Predict Positive Screening of Methicillin-Resistant Staphylococcus aureus During Mechanical Ventilation Using Synthetic Dataset From MIMIC-IV Database[52]	Journal (Front Med)	IV	TRUE	FALSE	2021
Machine learning-based prediction of in-hospital mortality for critically ill patients with sepsis-associated acute kidney injury[53]	Journal (Ren Fail)	IV	TRUE	TRUE	2024
Explainable machine learning model for predicting furosemide responsiveness in	Journal (Ren Fail)	IV	TRUE	FALSE	2023

© 2025 Ramadan B et al. *JAMA Network Open*.

patients with oliguric acute kidney injury[54]					
Interpretable machine learning model for early prediction of 28-day mortality in ICU patients with sepsis-induced coagulopathy: development and validation[55]	Journal (Eur J Med Res)	IV	TRUE	TRUE	2024
Machine learning-based models for predicting mortality and acute kidney injury in critical pulmonary embolism[56]	Journal (BMC Cardiovasc Disord)	IV	TRUE	TRUE	2023
Predicting risk for trauma patients using static and dynamic information from the MIMIC III database[57]	Journal (PLoS One)	III	TRUE	FALSE	2022
Machine learning-based mortality prediction model for critically ill cancer patients admitted to the intensive care unit (CanICU)[58]	Journal (Cancers)	III	TRUE	FALSE	2023
Prediction of acute kidney injury in patients with liver cirrhosis using machine learning models: evidence from the MIMIC-III and MIMIC-IV[59]	Journal (Int Urol Nephrol)	III & IV	TRUE	FALSE	2024
Machine learning-based prediction model of acute kidney injury in patients with acute respiratory distress syndrome[60]	Journal (BMC Pulm Med)	III & IV	TRUE	TRUE	2023
Early prediction of MODS interventions in the intensive care unit using machine learning[61]	Journal (J Big Data)	III & IV	TRUE	FALSE	2023
Prediction of in-hospital mortality of intensive care unit patients with acute pancreatitis based on an explainable machine learning algorithm[62]	Journal (J Clin Gastroenterol)	IV	TRUE	FALSE	2024
Prediction of in-hospital mortality for ICU patients with heart failure[63]	Preprint (medRxiv)	III	TRUE	TRUE	2024
Practical machine learning-based sepsis prediction[64]	Conference (IEEE)	III	TRUE	FALSE	2020
30-day hospital readmission prediction using MIMIC data[65]	Conference (IEEE)	III	FALSE	TRUE	2020
A Retrospective cohort study: predicting 90-day mortality for ICU trauma patients with a machine learning algorithm using XGBoost using MIMIC-III database[66]	Journal (J Multidiscip Healthc)	III	FALSE	TRUE	2023
A predictive model for the risk of sepsis within 30 days of admission in patients with traumatic brain injury in the intensive care unit: a retrospective analysis[67]	Journal (Eur J Med Res)	III & IV	TRUE	FALSE	2023
Prostate cancer prediction model: a retrospective analysis based on machine learning using the MIMIC-IV database[68]	Journal (Intelligent Pharmacy)	IV	TRUE	TRUE	2023
Predictive model of acute kidney injury in critically ill patients with acute pancreatitis: a machine learning approach using the MIMIC-IV database[69]	Journal (Ren Fail)	IV	TRUE	TRUE	2024
A Machine Learning pipeline using KNIME to predict hospital admission in the MIMIC-IV Database[70]	Conference (IEEE)	IV	TRUE	FALSE	2023
Comparison of machine learning algorithms for mortality prediction in intensive care patients on multi-center critical care databases[71]	Journal (IOP Conf Ser Mater Sci Eng)	III & IV	TRUE	FALSE	2021
Predicting in-hospital mortality for MIMIC-III patients: A nomogram combined with SOFA score[72]	Journal (Medicine)	III	TRUE	TRUE	2022

© 2025 Ramadan B et al. *JAMA Network Open*.

Machine Learning Model for the Prediction of Hemorrhage in Intensive Care Units[73]	Journal (Healthc Inform Res)	III & IV	TRUE	FALSE	2022
Prediction of 30-day mortality for ICU patients with Sepsis-3[74]	Journal (BMC Med Inform Decis Mak)	III	TRUE	FALSE	2024
Development and validation of a machine-learning model for prediction of hypoxemia after extubation in intensive care units[75]	Journal (Ann Transl Med)	IV	TRUE	TRUE	2022
Machine learning as a tool to identify inpatients who are not at risk of adverse drug events in a large dataset of a tertiary care hospital in the USA[76]	Journal (Br J Clin Pharmacol)	IV	TRUE	FALSE	2023
Development and validation of a machine-learning model for predicting the risk of death in sepsis patients with acute kidney injury[77]	Journal (Heliyon)	IV	TRUE	TRUE	2024
Survival Prediction in Patients with Hypertensive Chronic Kidney Disease in Intensive Care Unit: A Retrospective Analysis Based on the MIMIC[78] Database	Journal (J Immunol Res)	III	FALSE	TRUE	2022
On the early detection of Sepsis in MIMIC-III[79]	Conference (IEEE)	III	TRUE	FALSE	2021
Comparison of machine learning algorithms to SAPS II in predicting in-hospital mortality of fractures of the pelvis and acetabulum: analyzes based on MIMIC-III database[80]	Journal (All Life)	III	TRUE	FALSE	2022
Predicting Mortality in Sepsis-Associated Acute Respiratory Distress Syndrome: A Machine Learning Approach using the MIMIC-III database[81]	Journal (Journal of Intensive Care Medicine)	III	TRUE	FALSE	2024
Explainable Machine Learning Models for Pneumonia Mortality Risk Prediction Using MIMIC-III Data[82]	Conference (Int. Conf. Soft Comput. Mach. Intell)	III	TRUE	TRUE	2022
Simplified &Novel Predictive Model using Feature Engineering over MIMIC-III Dataset[83]	Journal (Procedia Comput Sci)	III	TRUE	TRUE	2023
XGBoost in the Prediction of 28-Day Mortality in Critical Elderly Patients with Hip Fracture: A MIMIC-IV Cohort Study[84]	Journal (Altern Ther Health Med)	IV	TRUE	TRUE	2024
Predicting Prescribed Medications from the MIMIC-IV Event and Measurement Data[85]	Thesis	IV	TRUE	FALSE	2023
Clinical nomogram prediction model to assess the risk of prolonged ICU length of stay in patients with diabetic ketoacidosis: a retrospective analysis based on the MIMIC-IV database[86]	Journal (BMC Anesthesiol)	IV	TRUE	FALSE	2024
Internal and external validation of machine learning–assisted prediction models for mechanical ventilation–associated severe acute kidney injury[87]	Journal (Aust Crit Care)	IV	TRUE	TRUE	2023
Nomogram establishment for short-term survival prediction in ICU patients with aplastic anemia based on the MIMIC-IV database[88]	Journal (Hematology)	IV	TRUE	TRUE	2024
Predicting patient outcome from clinical journals and biomedical articles: Using the MIMIC-IV database, multiple in-hospital mortality prediction models are created, to which improvements are	Thesis	IV	TRUE	FALSE	2022

© 2025 Ramadan B et al. *JAMA Network Open*.

attempted through the use of word embeddings trained on scientific biomedical literature[89]					
Machine learning time-to-event mortality prediction in MIMIC-IV critical care database[90]	Thesis	IV	TRUE	FALSE	2022
Machine learning models to predict 30-day mortality for critical patients with myocardial infarction: a retrospective analysis from MIMIC-IV database[91]	Journal (Front Cardiovasc Med)	IV	TRUE	NA	2024
ML-Based AKI Prediction in Acute Pancreatitis: Innovative Models from MIMIC-IV Database[92]	Journal (Ren. Fail)	IV	TRUE	TRUE	2024
Exploring Predictive Factors for Heart Failure Progression in Hypertensive Patients Based on Medical Diagnosis Data from the MIMIC-IV Database[93]	Journal (Bioengineering)	IV	FALSE	TRUE	2024
A novel clinical prediction model for in-hospital mortality in sepsis patients complicated by ARDS: A MIMIC IV database and external validation study[94]	Journal (Heliyon)	IV	TRUE	TRUE	2024
Factors and machine learning models for predicting successful discontinuation of continuous renal replacement therapy in critically ill patients with acute kidney injury: a retrospective cohort study based on MIMIC-IV database[95]	Journal (BMC Nephrol)	IV	TRUE	NA	2024
Machine learning for in-hospital mortality prediction in critically ill patients with acute heart failure: A retrospective analysis based on MIMIC-IV databases[96]	Journal (J. Cardiothorac. Vasc. Anesth)	IV	TRUE	TRUE	2025
Evaluating the Fairness of the MIMIC-IV Dataset and a Baseline Algorithm: Application to the ICU Length of Stay Prediction[97]	Preprint (arXiv)	IV	TRUE	NA	2023
Multitask learning to predict successful weaning in critically ill ventilated patients: A retrospective analysis of the MIMIC-IV database[98]	Journal (Digit Health)	IV	TRUE	NA	2024
Development of machine learning models for predicting acute respiratory distress syndrome: evidence from the MIMIC-III and MIMIC-IV[99]	Preprint (Research Square)	III & IV	TRUE	NA	2023
Feature selection and risk prediction for diabetic patients with ketoacidosis based on MIMIC-IV[100]	Journal (Front Endocrinol)	IV	TRUE	TRUE	2024

© 2025 Ramadan B et al. *JAMA Network Open*.

eFigure. Calibration Curves and Predicted Distributions for Trained Classifiers. For the three classifiers trained to predict inpatient mortality from same-admission ICD codes, we present (top) calibration curves and (bottom) histograms of the predicted probabilities of mortality on the test set. Note that the propensity for the random forest classifier to predict values away from 0 and 1 is expected behavior. As explained by Niculescu-Mizil and Caruana,[101] "Methods such as bagging and random forests that average predictions from a base set of models can have difficulty making predictions near 0 and 1 because variance in the underlying base models will bias predictions that should be near zero or one away from these values. Because predictions are restricted to the interval [0,1], errors caused by variance tend to be one-sided near zero and one. For example, if a model should predict p=0 for a case, the only way bagging can achieve this is if all bagged trees predict zero." Separate to the properties inherent to ensemble methods, the relatively poor calibration is likely to be an artifact of the class imbalance as well as potential further evidence of the inappropriateness of models relying on label leakage for deployment.

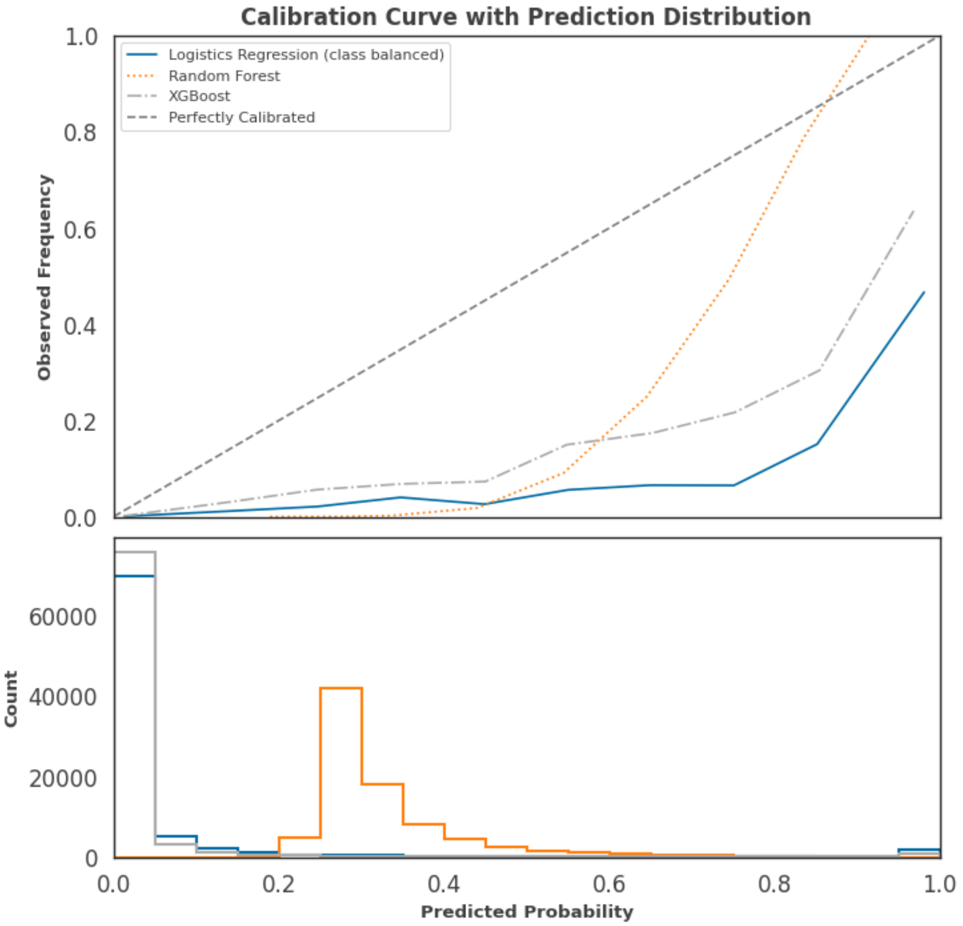

© 2025 Ramadan B et al. *JAMA Network Open*.

eReferences

1. Hou N, Li M, He L, et al. Predicting 30-days mortality for MIMIC-III patients with sepsis-3: a machine learning approach using XGboost. *J Transl Med*. 2020;18(1):462.
2. Li F, Xin H, Zhang JD, Fu M, Zhou J, Lian Z. Prediction model of in-hospital mortality in intensive care unit patients with heart failure: machine learning-based, retrospective analysis of the MIMIC-III database. *BMJ Open*. 2021;11. doi:10.1136/bmjopen-2020-044779
3. Scherpf M, Gräßer F, Malberg H, Zaunseder S. Predicting sepsis with a recurrent neural network using the MIMIC III database. *Comput Biol Med*. 2019;113(103395):103395.
4. Bao C, Deng F, Zhao S. Machine-learning models for prediction of sepsis patients mortality. *Med Intensiva (Engl Ed)*. 2023;47(6):315-325.
5. Zhong Z, Yuan X, Liu S, Yang Y, Liu F. Machine learning prediction models for prognosis of critically ill patients after open-heart surgery. *Sci Rep*. 2021;11(1):3384.
6. Lei M, Han Z, Wang S, et al. A machine learning-based prediction model for in-hospital mortality among critically ill patients with hip fracture: An internal and external validated study. *Injury*. 2023;54(2):636-644.
7. Zhu Y, Zhang J, Wang G, et al. Machine learning prediction models for mechanically ventilated patients: Analyses of the MIMIC-III database. *Front Med (Lausanne)*. 2021;8:662340.
8. McWilliams CJ, Lawson DJ, Santos-Rodriguez R, et al. Towards a decision support tool for intensive care discharge: machine learning algorithm development using electronic healthcare data from MIMIC-III and Bristol, UK. *BMJ Open*. 2019;9(3):e025925.
9. Zhao QY, Wang H, Luo JC, et al. Development and validation of a machine-learning model for prediction of extubation failure in Intensive Care units. *Front Med (Lausanne)*. 2021;8:676343.
10. Xie F, Zhou J, Lee JW, et al. Benchmarking emergency department prediction models with machine learning and public electronic health records. *Sci Data*. 2021;9. doi:10.1038/s41597-022-01782-9
11. Hu C, Tan Q, Zhang Q, et al. Application of interpretable machine learning for early prediction of prognosis in acute kidney injury. *Comput Struct Biotechnol J*. 2022;20:2861-2870.
12. Wang Z, Zhang L, Huang T, et al. Developing an explainable machine learning model to predict the mechanical ventilation duration of patients with ARDS in intensive care units. *Heart Lung*. 2023;58:74-81.
13. Nistal-Nuño B. Developing machine learning models for prediction of mortality in the medical intensive care unit. *Comput Methods Programs Biomed*. 2022;216(106663):106663.
14. Bendavid I, Statlender L, Shvartser L, et al. A novel machine learning model to predict respiratory failure and invasive mechanical ventilation in critically ill patients suffering from COVID-19. *Sci Rep*. 2022;12(1):10573.
15. Gentimis T, Alnaser AJ, Durante A, Cook K, Steele R. Predicting hospital length of stay using neural networks on MIMIC III data. In: *2017 IEEE 15th Intl Conf on Dependable, Autonomic and Secure Computing, 15th Intl Conf on Pervasive Intelligence and Computing, 3rd Intl Conf on Big Data Intelligence and Computing and Cyber Science and Technology Congress(DASC/PiCom/DataCom/CyberSciTech)*. IEEE; 2017:1194-1201.
16. Peng X, Li L, Wang X, Zhang H. A machine learning-based prediction model for acute kidney injury in patients with congestive heart failure. *Front Cardiovasc Med*. 2022;9:842873.
17. Hu C, Li L, Li Y, Wang F, Hu B, Peng Z. Explainable machine-learning model for prediction of in-hospital mortality in septic patients requiring Intensive Care unit readmission. *Infect Dis Ther*. 2022;11(4):1695-1713.
18. Bashar SK, Hossain MB, Ding E, Walkey AJ, McManus DD, Chon KH. Atrial fibrillation detection during sepsis: Study on MIMIC III ICU data. *IEEE J Biomed Health Inform*. 2020;24(11):3124-3135.
19. Zhao QY, Liu LP, Luo JC, et al. A machine-learning approach for dynamic prediction of sepsis-induced coagulopathy in critically ill patients with sepsis. *Front Med (Lausanne)*. 2020;7:637434.
20. Hempel L, Sadeghi S, Kirsten T. Prediction of intensive care unit length of stay in the MIMIC-IV dataset. *Appl Sci (Basel)*. 2023;13(12):6930.
21. Liang Y, Zhu C, Tian C, et al. Early prediction of ventilator-associated pneumonia in critical care patients: a machine learning model. *BMC Pulm Med*. 2022;22(1):250.
22. Sayed M, Riaño D, Villar J. Predicting duration of mechanical ventilation in acute respiratory distress syndrome using supervised machine learning. *J Clin Med*. 2021;10(17):3824.

© 2025 Ramadan B et al. *JAMA Network Open*.

23. Hasan MN, Hamdan S, Poudel S, Vargas J, Poudel K. Prediction of length-of-stay at intensive care unit (ICU) using machine learning based on MIMIC-III database. In: *2023 IEEE Conference on Artificial Intelligence (CAI)*. IEEE; 2023:321-323.
24. Beaulieu-Jones BK, Orzechowski P, Moore JH. Mapping Patient Trajectories using Longitudinal Extraction and Deep Learning in the MIMIC-III Critical Care Database. *Pac Symp Biocomput*. Published online 2017:177428.
25. Xie W, Li Y, Meng X, Zhao M. Machine learning prediction models and nomogram to predict the risk of in-hospital death for severe DKA: A clinical study based on MIMIC-IV, eICU databases, and a college hospital ICU. *Int J Med Inform*. 2023;174(105049):105049.
26. Zhang Y, Hu J, Hua T, Zhang J, Zhang Z, Yang M. Development of a machine learning-based prediction model for sepsis-associated delirium in the intensive care unit. *Sci Rep*. 2023;13(1):12697.
27. Wang B, Li Y, Tian Y, Ju C, Xu X, Pei S. Novel pneumonia score based on a machine learning model for predicting mortality in pneumonia patients on admission to the intensive care unit. *Respir Med*. 2023;217(107363):107363.
28. Tang F, Xiao C, Wang F, Zhou J. Predictive modeling in urgent care: a comparative study of machine learning approaches. *JAMIA Open*. 2018;1(1):87-98.
29. Lu Z, Zhang J, Hong J, et al. Development of a nomogram to predict 28-day mortality of patients with sepsis-induced coagulopathy: An analysis of the MIMIC-III database. *Front Med (Lausanne)*. 2021;8:661710.
30. Huang B, Liang D, Zou R, et al. Mortality prediction for patients with acute respiratory distress syndrome based on machine learning: a population-based study. *Ann Transl Med*. 2021;9(9):794.
31. Camacho-Cogollo JE, Bonet I, Gil B, Iadanza E. Machine learning models for early prediction of sepsis on large healthcare datasets. *Electronics (Basel)*. 2022;11(9):1507.
32. Danilatou V, Nikolakakis S, Antonakaki D, et al. Outcome prediction in critically-ill patients with venous thromboembolism and/or cancer using machine learning algorithms: External validation and comparison with scoring systems. *Int J Mol Sci*. 2022;23(13):7132.
33. Zhao Y, Zhang R, Zhong Y, et al. Statistical analysis and machine learning prediction of disease outcomes for COVID-19 and pneumonia patients. *Front Cell Infect Microbiol*. 2022;12:838749.
34. Sun Y, He Z, Ren J, Wu Y. Prediction model of in-hospital mortality in intensive care unit patients with cardiac arrest: a retrospective analysis of MIMIC -IV database based on machine learning. *BMC Anesthesiol*. 2023;23(1):178.
35. Shu T, Huang J, Deng J, et al. Development and assessment of scoring model for ICU stay and mortality prediction after emergency admissions in ischemic heart disease: a retrospective study of MIMIC-IV databases. *Intern Emerg Med*. 2023;18(2):487-497.
36. Ning YL, Sun C, Xu XH, et al. Tendency of dynamic vasoactive and inotropic medications data as a robust predictor of mortality in patients with septic shock: An analysis of the MIMIC-IV database. *Front Cardiovasc Med*. 2023;10:1126888.
37. Budrionis A, Miara M, Miara P, Wilk S, Bellika JG. Benchmarking PySyft federated learning framework on MIMIC-III dataset. *IEEE Access*. 2021;9:116869-116878.
38. Tang H, Jin Z, Deng J, et al. Development and validation of a deep learning model to predict the survival of patients in ICU. *J Am Med Inform Assoc*. 2022;29(9):1567-1576.
39. Pang K, Li L, Wen O, Liu X, Tang Y. Establishment of ICU mortality risk prediction models with machine learning algorithm using MIMIC-IV database. *Diagnostics (Basel)*. 2022;12. doi:10.3390/diagnostics12051068
40. Zeng Z, Yao S, Zheng J, Gong X. Development and validation of a novel blending machine learning model for hospital mortality prediction in ICU patients with Sepsis. *BioData Min*. 2021;14(1):40.
41. Wang R, Cai L, Liu Y, Zhang J, Ou X, Xu J. Machine learning algorithms for prediction of ventilator associated pneumonia in traumatic brain injury patients from the MIMIC-III database. *Heart Lung*. 2023;62:225-232.
42. Liu F, Yao J, Liu C, Shou S. Construction and validation of machine learning models for sepsis prediction in patients with acute pancreatitis. *BMC Surg*. 2023;23(1):267.
43. Hur S, Ko RE, Yoo J, Ha J, Cha WC, Chung CR. A machine learning-based algorithm for the prediction of intensive care unit delirium (PRIDE): Retrospective study. *JMIR Med Inform*. 2021;9(7):e23401.

© 2025 Ramadan B et al. *JAMA Network Open*.

44. Huang AA, Huang SY. Dendrogram of transparent feature importance machine learning statistics to classify associations for heart failure: A reanalysis of a retrospective cohort study of the Medical Information Mart for Intensive Care III (MIMIC-III) database. *PLoS One*. 2023;18(7):e0288819.
45. Yang W, Zou H, Wang M, Zhang Q, Li S, Liang H. Mortality prediction among ICU inpatients based on MIMIC-III database results from the conditional medical generative adversarial network. *Heliyon*. 2023;9(2):e13200.
46. Zhang X, Fei N, Zhang X, Wang Q, Fang Z. Machine learning prediction models for postoperative stroke in elderly patients: Analyses of the MIMIC database. *Front Aging Neurosci*. 2022;14:897611.
47. Su Y, Guo C, Zhou S, Li C, Ding N. Early predicting 30-day mortality in sepsis in MIMIC-III by an artificial neural networks model. *Eur J Med Res*. 2022;27(1):294.
48. Chang HH, Chiang JH, Wang CS, et al. Predicting mortality using machine learning algorithms in patients who require renal replacement therapy in the critical care unit. *J Clin Med*. 2022;11(18):5289.
49. Liu W, Tao G, Zhang Y, et al. A simple weaning model based on interpretable machine learning algorithm for patients with sepsis: A research of MIMIC-IV and eICU databases. *Front Med (Lausanne)*. 2021;8:814566.
50. Nowroozilarki Z, Pakbin A, Royalty J, Lee DKK, Mortazavi BJ. Real-time mortality prediction using MIMIC-IV ICU data via boosted nonparametric hazards. In: *2021 IEEE EMBS International Conference on Biomedical and Health Informatics (BHI)*. IEEE; 2021:1-4.
51. Khope SR, Elias S. Critical correlation of predictors for an efficient risk prediction framework of ICU patient using correlation and transformation of MIMIC-III dataset. *Data Sci Eng*. 2022;7(1):71-86.
52. Hirano Y, Shinmoto K, Okada Y, et al. Machine learning approach to predict positive screening of methicillin-resistant Staphylococcus aureus during mechanical ventilation using synthetic dataset from MIMIC-IV database. *Front Med (Lausanne)*. 2021;8:694520.
53. Gao T, Nong Z, Luo Y, et al. Machine learning-based prediction of in-hospital mortality for critically ill patients with sepsis-associated acute kidney injury. *Ren Fail*. 2024;46(1):2316267.
54. Jiang M, Pan CQ, Li J, Xu LG, Li CL. Explainable machine learning model for predicting furosemide responsiveness in patients with oliguric acute kidney injury. *Ren Fail*. 2023;45(1):2151468.
55. Zhou S, Lu Z, Liu Y, et al. Interpretable machine learning model for early prediction of 28-day mortality in ICU patients with sepsis-induced coagulopathy: development and validation. *Eur J Med Res*. 2024;29(1):14.
56. Wang G, Xu J, Lin X, et al. Machine learning-based models for predicting mortality and acute kidney injury in critical pulmonary embolism. *BMC Cardiovasc Disord*. 2023;23(1):385.
57. Tsiklidis EJ, Sinno T, Diamond SL. Predicting risk for trauma patients using static and dynamic information from the MIMIC III database. *PLoS One*. 2022;17(1):e0262523.
58. Ko RE, Cho J, Shin MK, et al. Machine learning-based mortality prediction model for critically ill cancer patients admitted to the Intensive Care unit (CanICU). *Cancers (Basel)*. 2023;15(3):569.
59. Tian J, Cui R, Song H, Zhao Y, Zhou T. Prediction of acute kidney injury in patients with liver cirrhosis using machine learning models: evidence from the MIMIC-III and MIMIC-IV. *Int Urol Nephrol*. 2024;56(1):237-247.
60. Wei S, Zhang Y, Dong H, et al. Machine learning-based prediction model of acute kidney injury in patients with acute respiratory distress syndrome. *BMC Pulm Med*. 2023;23(1):370.
61. Liu C, Yao Z, Liu P, et al. Early prediction of MODS interventions in the intensive care unit using machine learning. *J Big Data*. 2023;10(1):55.
62. Ren W, Zou K, Huang S, et al. Prediction of in-hospital mortality of Intensive Care unit patients with acute pancreatitis based on an explainable machine learning algorithm. *J Clin Gastroenterol*. 2024;58(6):619-626.
63. Zhang J, Li H, Ashrafi N, Yu Z, Placencia G, Pishgar M. Prediction of in-hospital mortality for ICU patients with heart failure. *medRxiv*. Published online June 25, 2024. doi:10.1101/2024.06.25.24309448
64. Pettinati MJ, Chen G, Rajput KS, Selvaraj N. Practical machine learning-based sepsis prediction. *Annu Int Conf IEEE Eng Med Biol Soc*. 2020;2020:4986-4991.
65. Assaf R, Jayousi R. 30-day Hospital Readmission Prediction using MIMIC Data. In: *2020 IEEE 14th International Conference on Application of Information and Communication Technologies (AICT)*. IEEE; 2020:1-6.

© 2025 Ramadan B et al. *JAMA Network Open*.

66. Yang S, Cao L, Zhou Y, Hu C. A retrospective cohort study: Predicting 90-day mortality for ICU trauma patients with a machine learning algorithm using XGBoost using MIMIC-III database. *J Multidiscip Healthc*. 2023;16:2625-2640.
67. Hu F, Zhu J, Zhang S, et al. A predictive model for the risk of sepsis within 30 days of admission in patients with traumatic brain injury in the intensive care unit: a retrospective analysis based on MIMIC-IV database. *Eur J Med Res*. 2023;28(1):290.
68. Wang W, Jin X. Prostate cancer prediction model: A retrospective analysis based on machine learning using the MIMIC-IV database. *Intelligent Pharmacy*. 2023;1(4):268-273.
69. Lin S, Lu W, Wang T, et al. Predictive model of acute kidney injury in critically ill patients with acute pancreatitis: a machine learning approach using the MIMIC-IV database. *Ren Fail*. 2024;46(1):2303395.
70. Tsoni R, Kaldis V, Kapogianni I, Sakagianni A, Feretzakis G, Verykios VS. A machine learning pipeline using KNIME to predict hospital admission in the MIMIC-IV database. In: *2023 14th International Conference on Information, Intelligence, Systems & Applications (IISA)*. IEEE; 2023:1-6.
71. Pattalung TN, Chaichulee S. Comparison of machine learning algorithms for mortality prediction in intensive care patients on multi-center critical care databases. *IOP Conf Ser Mater Sci Eng*. 2021;1163(1):012027.
72. Liu R, Liu H, Li L, Wang Z, Li Y. Predicting in-hospital mortality for MIMIC-III patients: A nomogram combined with SOFA score. *Medicine (Baltimore)*. 2022;101. doi:10.1097/MD.0000000000031251
73. Kang S, Park C, Lee J, Yoon D. Machine learning model for the prediction of hemorrhage in Intensive Care units. *Healthc Inform Res*. 2022;28(4):364-375.
74. Yu Z, Ashrafi N, Li H, Alaei K, Pishgar M. Prediction of 30-day mortality for ICU patients with Sepsis-3. *BMC Med Inform Decis Mak*. 2024;24(1):223.
75. Xia M, Jin C, Cao S, et al. Development and validation of a machine-learning model for prediction of hypoxemia after extubation in intensive care units. *Ann Transl Med*. 2022;10(10):577.
76. Langenberger B. Machine learning as a tool to identify inpatients who are not at risk of adverse drug events in a large dataset of a tertiary care hospital in the USA. *Br J Clin Pharmacol*. 2023;89(12):3523-3538.
77. Dong L, Liu P, Qi Z, Lin J, Duan M. Development and validation of a machine-learning model for predicting the risk of death in sepsis patients with acute kidney injury. *Heliyon*. 2024;10(9):e29985.
78. Xia Z, Xu P, Xiong Y, Lai Y, Huang Z. Survival prediction in patients with hypertensive chronic kidney disease in Intensive Care Unit: A retrospective analysis based on the MIMIC-III database. *J Immunol Res*. 2022;2022:3377030.
79. Medina M, Sala P. On the early detection of Sepsis in MIMIC-III. In: *2021 IEEE 9th International Conference on Healthcare Informatics (ICHI)*. IEEE; 2021:171-180.
80. Jiang X, Dai W, Cai Y. Comparison of machine learning algorithms to SAPS II in predicting in-hospital mortality of fractures of the pelvis and acetabulum: analyzes based on MIMIC-III database. All Life. 2022 Sep 22;15(1):1000–12.
81. Mu S, Yan D, Tang J, Zheng Z. Predicting Mortality in Sepsis-Associated Acute Respiratory Distress Syndrome: A Machine Learning Approach Using the MIMIC-III Database. Journal of Intensive Care Medicine. 2024 Sep 5;
82. Sanii J, Wai Yip Chan. Explainable Machine Learning Models for Pneumonia Mortality Risk Prediction Using MIMIC-III Data. ; *Int. Conf. Soft Comput. Mach. Intell.* 2022.
83. Khope SR, Elias S. Simplified & novel predictive model using feature engineering over MIMIC-III dataset. *Procedia Comput Sci*. 2023;218:1968-1976.
84. Yuan W, Xiao M, Wang R, Liu G, Wu J, Wang X. XGBoost in the prediction of 28-day mortality in critical elderly patients with hip fracture: A MIMIC-IV cohort study. *Altern Ther Health Med*. 2024;30(9): 432-436.
85. Reponen M. Predicting Prescribed Medications from the MIMIC-IV Event and Measurement Data. https://dspace.uef.fi/bitstream/handle/123456789/30580/urn_nbn_fi_uef-20231201.pdf?sequence=1&isAllowed=y
86. Shi J, Chen F, Zheng K, et al. Clinical nomogram prediction model to assess the risk of prolonged ICU length of stay in patients with diabetic ketoacidosis: a retrospective analysis based on the MIMIC-IV database. *BMC Anesthesiol*. 2024;24(1):86.
87. Huang S, Teng Y, Du J, Zhou X, Duan F, Feng C. Internal and external validation of machine learning-assisted prediction models for mechanical ventilation-associated severe acute kidney injury. *Aust Crit Care*. 2023;36(4):604-612.

© 2025 Ramadan B et al. *JAMA Network Open*.

88. Tu Y, Zhang J, Zhao M, He F. Nomogram establishment for short-term survival prediction in ICU patients with aplastic anemia based on the MIMIC-IV database. *Hematology*. 2024;29(1):2339778.
89. Henriksson F, Svensson, P. Predicting patient outcome from clinical journals and biomedical articles: Using the MIMIC-IV database, multiple in-hospital mortality prediction models are created, to …. https://odr.chalmers.se/items/a104afb1-3220-494c-b149-0a85b9d18117
90. Royalty JP. Machine Learning Time-to-Event Mortality Prediction in MIMIC-IV Critical Care Database. Published online July 24, 2021. Accessed January 28, 2025. https://hdl.handle.net/1969.1/194429
91. Lin X, Pan X, Yang Y, et al. Machine learning models to predict 30-day mortality for critical patients with myocardial infarction: a retrospective analysis from MIMIC-IV database. *Front Cardiovasc Med*. 2024;11:1368022.
92. Lin S, Lu W, Wang T, et al. Predictive model of acute kidney injury in critically ill patients with acute pancreatitis: a machine learning approach using the MIMIC-IV database. *Ren. Fail*. 2024;46(1).
93. Jung J, Kim D, Hwang I. Exploring predictive factors for heart failure progression in hypertensive patients based on medical diagnosis data from the MIMIC-IV database. *Bioengineering (Basel)*. 2024;11(6):531.
94. Chen Y, Zong C, Zou L, et al. A novel clinical prediction model for in-hospital mortality in sepsis patients complicated by ARDS: A MIMIC IV database and external validation study. *Heliyon*. 2024;10(13):e33337.
95. Sheng S, Li A, Liu X, et al. Factors and machine learning models for predicting successful discontinuation of continuous renal replacement therapy in critically ill patients with acute kidney injury: a retrospective cohort study based on MIMIC-IV database. *BMC Nephrol*. 2024;25(1):407.
96. Li J, Sun Y, Ren J, Wu Y, He Z. Machine learning for in-hospital mortality prediction in critically ill patients with acute heart failure: A retrospective analysis based on MIMIC-IV databases. J. Cardiothorac. Vasc. Anesth. 2025;39(3):666-674.
97. Kakadiaris A. Evaluating the fairness of the MIMIC-IV dataset and a baseline algorithm: Application to the ICU length of stay prediction. *arXiv [csLG]*. Published online December 31, 2023. http://arxiv.org/abs/2401.00902
98. Lin MY, Chi HY, Chao WC. Multitask learning to predict successful weaning in critically ill ventilated patients: A retrospective analysis of the MIMIC-IV database. *Digit Health*. 2024;10:20552076241289732.
99. Yang M, Hu W, Yan J. Development of machine learning models for predicting acute respiratory distress syndrome: evidence from the MIMIC-III and MIMIC-IV. Published online 2023. https://www.researchsquare.com/article/rs-3221576/latest
100. Liu Y, Mo W, Wang H, Shao Z, Zeng Y, Bi J. Feature selection and risk prediction for diabetic patients with ketoacidosis based on MIMIC-IV. *Front Endocrinol (Lausanne)*. 2024;15:1344277.
101. Niculescu-Mizil A, Caruana R. Predicting good probabilities with supervised learning. *ICML* 2005;625-632.

© 2025 Ramadan B et al. *JAMA Network Open*.
