# Supplementary material for: Diagnostic Codes in AI Prediction Models and Label Leakage of Same-Admission Clinical Outcomes
Source: JAMA Netw Open. 2025 Dec 26;8(12):e2550454. doi: 10.1001/jamanetworkopen.2025.50454 (PMC12743287; doi:10.1001/jamanetworkopen.2025.50454)
Supplement: Supplement 3. — Data Sharing Statement [file jamanetwopen-e2550454-s003.pdf]

Data Sharing Statement

Data

Data available: Yes
Data types: Deidentified participant data
How to access data: https://physionet.org/content/mimiciv/3.1/
When available: With publication

Supporting Documents

Document types: Statistical/analytic code
How to access documents: https://github.com/bbj-lab/data-leakage
When available: With publication

Additional Information

Who can access the data: Data are available via Physionet.
Types of analyses: All source code for this work are available in a public github repository.
Mechanisms of data availability: Data are available via Physionet's data access agreement (not managed by investigators).
